# Supplementary material for: Early detection and counselling intervention of asthma symptoms in preschool children: study design of a cluster randomised controlled trial
Source: BMC Public Health. 2010 Sep 15;10:555. doi: 10.1186/1471-2458-10-555 (PMC2944378; doi:10.1186/1471-2458-10-555)
Supplement: Additional file 1 — Early detection tool for early detection of asthma symptoms in preschool children. The file contains the early detection tool (consisting of 6 questions). [file 1471-2458-10-555-S1.DOC]

## Additional file 1 - Early detection tool for early detection of asthma symptoms in preschool children.

**Early detection tool**

1. Has your child had wheezing or a whistling noise in the chest during the past 12 months?

□ Unknown

□ No

□ Yes, 1 or 2 times

□ Yes, 3 times or more

2. Has your child had wheezing or a whistling noise in the chest during the past 4 weeks?

□ Unknown

□ No

□ Yes, 1 or 2 times

□ Yes, 3 times or more

3. Has your child had shortness of breath or dyspnea during the past 12 months?

□ Unknown

□ No

□ Yes, 1 or 2 times

□ Yes, 3 times or more

4. Has your child had shortness of breath or dyspnea during the past 4 weeks?

□ Unknown

□ No

□ Yes, 1 or 2 times

□ Yes, 3 times or more

5. Has your child been treated by a general practitioner or paediatrician because of the above-mentioned symptoms (asthma therapy) during the past 4 weeks?

□ Unknown

□ No

□ Yes, the name of the medication is:…………………..

6. Has your child been exposed to tobacco smoke?

□ Unknown

□ No

□ Yes, sometimes

□ Yes, on a regular basis

□ Yes, often or daily
