# Supplementary material for: Early detection and counselling intervention of asthma symptoms in preschool children: study design of a cluster randomised controlled trial
Source: BMC Public Health. 2010 Sep 15;10:555. doi: 10.1186/1471-2458-10-555 (PMC2944378; doi:10.1186/1471-2458-10-555)
Supplement: Additional file 2 — Counselling intervention scheme following early detection of asthma symptoms (I) and tobacco smoke exposure (II). The file contains an overview of the steps of counselling intervention, following early detection of asthma symptoms (I) and tobacco smoke exposure (II) in preschool children. [file 1471-2458-10-555-S2.DOC]

Additional file 2 – Counselling intervention scheme following early detection of asthma symptoms (I) and tobacco smoke exposure (II).

I - Intervention scheme following detection of asthma symptoms in preschool children

Advice:

Visit the general practitioner when asthma symptoms return

No

Yes

*Presence of wheezing or shortness of breath or dyspnea during the past 12 months?*

1 or 2 episodes

3 or more episodes

No

No

Yes

Referral to general

practitioner

Referral to asthma nurse

Asthma information leaflet

*Treatment during the past 4 weeks (e.g. inhalation therapy)?*

*Presence of wheezing or shortness of breath or dyspnea during the past 4 weeks?*

No

Yes

II - Intervention scheme following detection of preschool child’s tobacco smoke exposure

*Child’s tobacco smoke exposure*

Counselling: prevent tobacco smoke exposure to the child supported by an information leaflet

No

Yes
